# A cross-sectional study of anxiety and depression caseness in female competitive figure skaters in Sweden

**DOI:** 10.1136/bmjsem-2022-001491

**Published:** 2023-03-08

**Authors:** Moa Jederström, Sara Agnafors, Christina L Ekegren, Kristina Fagher, Håkan Gauffin, Laura Korhonen, Jennifer Park, Armin Spreco, Toomas Timpka

**Affiliations:** 1 Athletics Research Center, Department of Health, Medicine and Caring Sciences, Division of Society and Health, Linköping University, Linköping, Sweden; 2 Department of Biomedical and Clinical Sciences, Division of Children’s and Women’s Health, Linköping University, Linköping, Sweden; 3 Department of Research, Södra Älvsborgs Hospital, Borås, Sweden; 4 Rehabilitation, Ageing and Independent Living (RAIL) Research Centre, School of Primary Allied Health Care, Monash University, Melbourne, Victoria, Australia; 5 Department of Health Sciences, Rehabilitation Medicine Research Group, Lund University, Lund, Sweden; 6 Department of Orthopaedics and Department of Biomedical and Clinical Sciences, Linköping University, Linkoping, Sweden; 7 Department of Child and Adolescent Psychiatry and Department of Biomedical and Clinical Sciences, Center for Social and Affective Neuroscience, Linköping University, Linkoping, Sweden; 8 Institute of Clinical Sciences, Department of Surgery, Sahlgrenska Academy, University of Gothenburg, Göteborg, Sweden; 9 Center for Health Services Development, Region Östergötland, Linköping, Sweden; 10 Department of Health, Medicine and Caring Sciences, Division of Society and Health, Linköping University, Linköping, Sweden

**Keywords:** figure skating, anxiety, depression, body image, epidemiology

## Abstract

**Objectives:**

Little is known about figure skaters’ mental health. This study aimed to describe anxiety and depression caseness (defined as a screening condition qualifying for psychiatric examination) in competitive figure skaters and analyse factors associated with such caseness.

**Methods:**

A cross-sectional study was performed in April 2019 among all competitive figure skaters in the south-eastern region of Sweden (N=400). The primary outcomes were anxiety caseness, measured using the short-form Spielberger State-Trait Anxiety Inventory and depression caseness, measured using the WHO-5 index. Multivariable logistic regression models were employed to determine the association between anxiety caseness and explanatory factors.

**Results:**

In total, 36% (n=142) of the invited skaters participated. Only females (n=137), mean age 12.9 (SD 3.0) years) were selected for analysis. Of the participating skaters, 47% displayed anxiety caseness and 10% depression caseness. Overweight body image perception (OR 5.9; 95% CI 2.0 to 17.6; p=0.001) and older age (OR 1.2; 95% CI 1.1 to 1.4; p=0.005) were associated with anxiety caseness. Skaters reporting no caseness were younger than those reporting only anxiety caseness (mean age difference −1.9 years; 95% CI −3.1 to −0.7; p=0.001) or anxiety and depression caseness (OR −3.5 years; 95% CI −5.6 to −1.5 years; p<0.001).

**Conclusion:**

Anxiety caseness was associated with overweight body image perception and older age in female competitive figure skaters. Older skaters reported generally worse mental health. More research on the mental health of figure skaters is warranted, considering comorbidity and focusing on those needing further assessment and support.

**WHAT IS ALREADY KNOWN ON THIS TOPIC**

- Figure skating is a popular sport among young females.
- Little is known about young figure skaters’ mental health.

**WHAT THIS STUDY ADDS**

- Overweight body image perception and older age were in female figure skaters associated with anxiety caseness.
- Older skaters reported worse mental health in anxiety and depression caseness.

**HOW THIS STUDY MIGHT AFFECT RESEARCH, PRACTICE OR POLICY**

- The findings indicate an association between body image perception and anxiety caseness among young female figure skaters, highlighting the need for early recognition of anxiety symptoms and early interventions to neutralise negative body image perceptions.

## Introduction

In parallel to its general positive effects, sports participation may put the athlete at risk of physical and mental ill health with an impact on overall well-being and performance.^1^ Physical and mental health are interrelated and jointly impact athletes’ well-being and performance. For example, anxiety has been found to predict sports injury risk in soccer players,^2^ and injured elite athletes express more anxiety and depressive symptoms.^3^ In some sports, specific associations have been reported. For instance, in elite athletics, women who have experienced sexual abuse are more likely to suffer from injuries^4^ and express suicidal ideation.^5^ Similarly, adolescent and adult aesthetic athletes, such as figure skaters, ballet dancers and rhythmic gymnasts, are at increased risk of developing a distinctive pattern of health problems, including anxiety, eating disorders and injuries.^6–9^

A recent study of young female Swedish figure skaters showed that older age and an increased number of skipped meals per week were associated with sustaining a sports injury episode.^10^ However, little is known about figure skaters’ mental health. The mean age of female figure skaters competing at a high level is lower than in most other Olympic sports.^11^ While risk factors for mental health issues have been studied to some extent in general youth populations, previous research on the mental health of young female figure skaters has included only small samples, and skaters are often grouped with other aesthetic athletes (eg, gymnasts and ballet dancers).^12^

To address these gaps in knowledge, the primary aim of this study was to describe the prevalence of anxiety and depression caseness (separately and in combination) in a representative, geographically defined Swedish population of licensed competitive figure skaters. The term caseness denotes a screening condition qualifying for psychiatric examination, that is, a notion of disease predisposition that warrants clinical assessment to prevent progression to significant pathology.^13^ The secondary aim was to examine determinants associated with anxiety and depression caseness. Among Swedish adolescents, older age has been reported to be positively associated with the rate of mental health complaints, especially among girls.^14^

## Methods

### Study design

This study employed a cross-sectional design. Data were collected using an online questionnaire to analyse sports injuries and assess mental health. The study follows the Strengthening the Reporting of Observational Studies in Epidemiology (STROBE) guidelines.

### Setting and participants

Licensed competitive skaters from all figure skating clubs in the Swedish South-eastern Regional Figure Skating Federation (part of the Swedish Figure Skating Association, which had a population of 5000 competitive skaters in the 2018–2019 season) were invited (N=400, age 5–31 years) to complete an online questionnaire. Clubs sent an email about the study and a link to the questionnaire to all guardians of skaters younger than 15 years old and all skaters 15 years or older. Participants received four email reminders during the response time. A previous article based on the same population that analysed sports injuries^10^ includes a detailed presentation of the data collection procedures and a non-response analysis. The sample was found to be representative according to sex and birthyear. Skaters from the lowest competitive level (star competitions level) were under-represented, skaters from the intermediate competitive level (club competitions level) were overrepresented and the highest competitive level (A-level and elite level) was representatively sampled.

#### Ethics Statement

Ethical approval was obtained from the Regional Ethics Committee in Linkoping, Sweden (Dnr 2018/483-31). The study follows the WMA Declaration of Helsinki Ethical Principles for Medical Research Involving Human Subjects. Informed written consent was obtained from all skaters and guardians of skaters younger than 15 years. The skaters could at any time withdraw their participation without stating a cause.

### Patient and public involvement statement

Former competitive figure skaters assisted with designing and testing the questionnaire. To test how younger skaters would perceive the questions, children not yet eligible for competition and their parents were also asked to test and evaluate the questionnaire during a pilot phase.

### Data collection

Data were collected in April–July 2019 through an online questionnaire (Lynes™). Clubs also contributed aggregated data (sex, date of birth (year and month) and competitive level) on the total population of their licensed competitive skaters.

Participating skaters completed different questionnaires based on age (</≥12 years). Parents of children <12 years of age were expected to help interpret questions if their children asked for help but to otherwise not oversee their children when participating. The web questionnaire was designed based on questionnaires previously used with athletics populations^15–18^ and within the 2014 study Health Behaviours in School-aged Children by the Public Health Agency of Sweden, on behalf of the WHO.^19^ Full details of the questionnaire have been previously published.^10^ In brief, the questionnaire took approximately 20 min to complete and included questions on skater characteristics and skating level, physical and mental health, and injury status.

### Outcome variables

Anxiety caseness (yes/no) was defined as a short-form Spielberger State-Trait Anxiety Inventory (short-STAI) score >12. Short-STAI is validated for adults^20^ and children aged 5–17 years.^21^ In the study in which short-STAI was developed, the authors converted short-STAI (6 questions scored 1–4; total score range 6–24) to a scale ranging from 20 to 80 for comparison with STAI-S (20 questions scored 1–4; total score range 20–80).^20^ In STAI-S, scores of 1–2 may indicate ‘no anxiety’, while scores of 3–4 may indicate ‘anxiety’. An STAI-S-cut-off score of >40 has been used in previous studies.^22 23^ This study used a corresponding cut-off score of >12 for short-STAI. By scoring >12, the responding athlete, on average, was less than moderately calm, relaxed and content and more than somewhat tense, upset and worried.

Depression caseness (yes/no) was defined as a WHO-5 score ≤40 for children between 9 and 12 years and ≤36 for adolescents between 13 and 16 years,^24^ and ≤50 for those ≥17 years.^25^ The depression caseness scores were calculated based on these age cut-offs for the WHO-5 instrument. The WHO-5 has been reported to be sensitive and specific when screening for depression.^25^ Each item is scored from zero (none of the time) to five (all of the time). In this study, the percentage score of the scale was used. The total index score ranges from the absence of well-being to the highest imaginable well-being.^25^

### Explanatory variables

Age (continuous) was self-reported by the skater, who stated the year and month of birth.

A severe sports injury episode (yes/no) was defined as any injury or pain that had occurred in connection with training or competition in figure skating that resulted in >21 days of lost or altered participation in figure skating.^26^

An ongoing sports injury episode (yes/no) was defined as a current injury or pain that had occurred in connection with training or competition in figure skating, which prevented the skater from fully participating in training or competition.

The number of skipped main meals per week (continuous) was indicated by combining the skater’s responses on how often they ate breakfast, lunch and dinner on weekdays and weekends, respectively.

Body mass index (BMI) was calculated from self-reports of weight and height, and for respondents <18 years of age presented as International Obesity Task Force-BMI (IOTF-BMI). This measure assesses underweight and overweight BMI values adjusted for age.^27^

The Syndrome of Relative Energy Deficiency in Sport (RED-S syndrome) (yes/no) was indicated if a skater reported irregular menstruation or was underweight, according to IOTF-BMI.

Body image perception (underweight/normal/overweight) was indicated based on the respondent’s categorisation of themselves.

‘Figure skating load’ (low/high) was used to attribute high skating load to those competing at the elite level or national level and/or being at a double loop or higher skating level.

Mean weekly training hours (≤6 hours/7–12 hours/≥13 hours) were indicated through self-report.

A dichotomous variable for parental education level (high/low) was created based on the skater having at least one parent who had completed postsecondary education (yes/no) and was used as an indicator of socioeconomic status.^28^

### Data analysis

All items related to skater characteristics, figure skating and determinants related to health status (physical and mental health) were presented descriptively using percentages for categorical variables and means and SD for continuous variables.

The low number of skaters reaching the cut-off level restricted the development of meaningful multiple models of determinants for depression caseness. Binary logistic regression analyses of determinants were therefore limited to anxiety caseness. Simple binary logistic regression analyses were initially used to identify explanatory factors associated with anxiety caseness. Then, a multivariable model was constructed by excluding the non-significant explanatory variables (p≥0.05) using backward elimination (Wald). Nagelkerke R^2^ was obtained for the multivariable model to estimate its accountability level. Associations with p<0.05 were considered to be statistically significant. All statistical tests were two sided. Three additional multivariable models were analysed as a sensitivity analysis, where significant variables were removed sequentially.

The analysis of factors associated with depression caseness was restricted to age and comorbidity with anxiety caseness. The skaters were classified into four groups based on their reported short-STAI and WHO-5 scores: (1) depression caseness and anxiety caseness, (2) depression caseness and no anxiety, (3) no depression and anxiety caseness and (4) neither depression nor anxiety caseness.

The age of participants in each group was compared with analysis of variance (ANOVA) with post-hoc Bonferroni correction.

All analyses were performed using The Statistical Package for the Social Sciences (SPSS) for Windows V.27.0.

## Results

**Figure 1:**
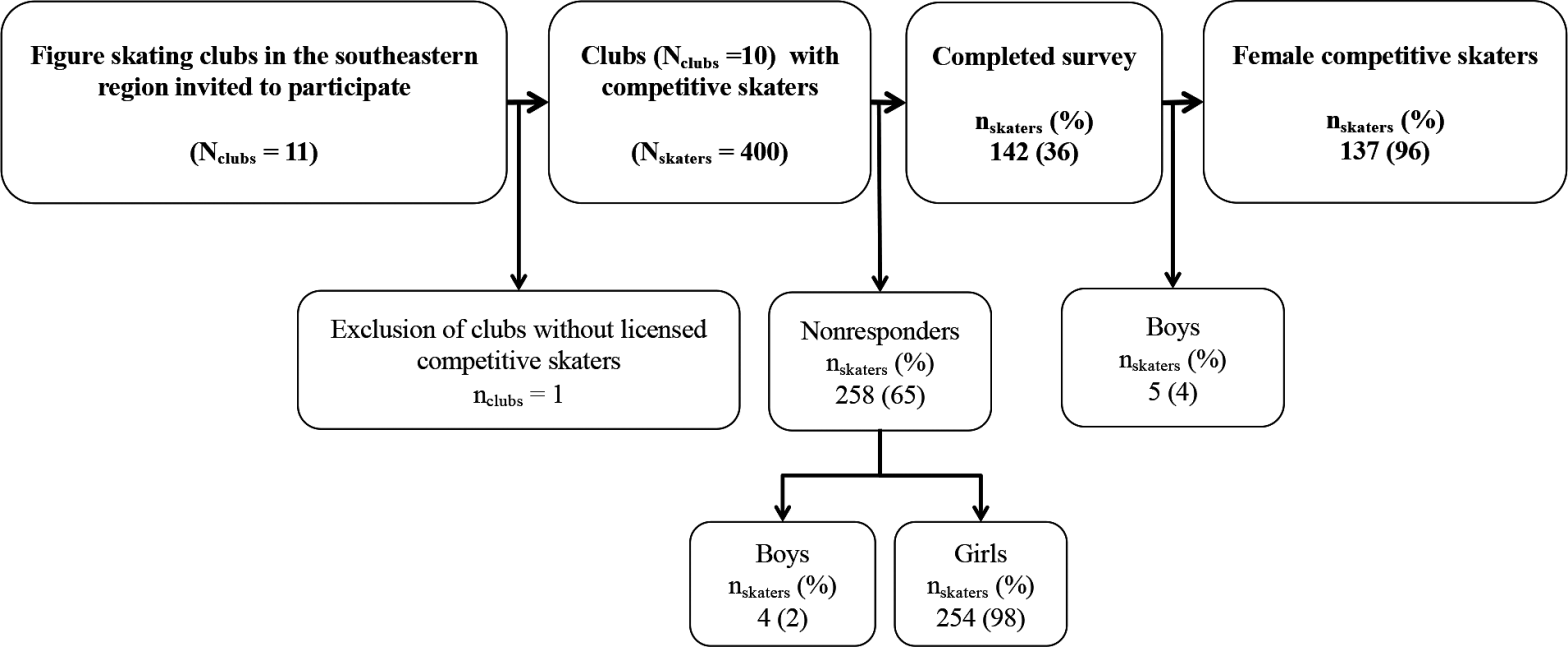
Study recruitment flow chart.

In total, 142 (36%) of the skaters invited participated; only girls (137 (96%)) were selected for analysis due to identification risk among boys (figure 1).

### Participant characteristics

The mean age of the participants was 12.9 (SD 3.0) years (range 6–23 years) (table 1). Most skaters’ parents were born in Sweden, and for the majority, at least one parent had completed postsecondary education. Most skaters lived with both guardians. Most participants ate breakfast, lunch and dinner every day. More than half of the participants exercised 7 hours per week or more outside school hours, with a majority practising figure skating between 4 and 9 hours each week. All participants were singles skaters, with 17% being elite skaters or skating at the national level (A-level) and 63% having landed double loop or more advanced jumps.

**Table 1:** Characteristics of participants and figure skating-specific data (n=137)

	Participants n (%)
Age	6–23 years
Mean (SD)	12.9 (3.0)
<12 years	47 (34)
12–15 years	68 (50)
>15 years	22 (16)
Parent’s country of origin	
Both parents born in Sweden	103 (75)
No parent born in Sweden	18 (13)
One parent born in Sweden	16 (12)
Parental education level	
At least one parent completed upper secondary education	15 (11)
At least one parent completed post-secondary education	122 (89)
Housing (n=136)	
Living with one guardian	9 (7)
Living with both guardians	120 (88)
Other (alternating between guardians, with a partner, etc)	7 (5)
Number of skipped main meals each week	
None	97 (71)
1–3 skipped main meals	22 (16)
4–6 skipped main meals	5 (4)
7–9 skipped main meals	7 (5)
>10 skipped main meals	6 (4)
Total exercise per week (days)	
Every day	7 (5)
4–6 days per week	100 (73)
≤3 days per week	30 (22)
Total exercise per week (hours)	
≤1 hour	5 (4)
2–3 hours	11 (8)
4–6 hours	44 (32)
7–9 hours	35 (26)
10–12 hours	28 (20)
≥13 hours	14 (10)
Figure skating per week (hours)	
2–3 hours	12 (9)
4–6 hours	67 (49)
7–9 hours	32 (23)
10–12 hours	19 (14)
≥13 hours	7 (5)
Resting days per week in the last 12 months (means)	
0 days	6 (4)
1–2 days	100 (73)
3–6 days	31 (23)
Discipline	
Singles skating	137 (100)
Competitive level	
Elite skater/A-competitions	23 (17)
Club competitions	61 (45)
Star competitions	53 (39)
Skating level	
Landed up to double toeloop	51 (37)
Landed double loop and higher	86 (63)

About one-third of the skaters had experienced a severe sports injury episode in the past year, and nearly one-fifth had an ongoing one.^10^ Most skaters rated their health as ‘excellent’ or ‘good’. According to self-reported data on height and weight, using IOTF-BMI, 12% were underweight and 4% were overweight. Regarding pubertal status, almost half had reached menarche, of whom 42% had irregular menstruation. Nearly one-third were careful not to gain weight, and one-fifth had a body image perception of being overweight. About a tenoth had been asked to gain weight (mainly by parents), and 7% had been asked to lose weight (mainly by coaches) (table 2, online supplemental table 1).

10.1136/bmjsem-2022-001491.supp1 Supplementary data

**Table 2:** Determinants related to health status in terms of physical and mental health (n=137)

	Participants n (%)
Severe injury episode in the last 12 months	
Yes	42 (31)
No	95 (69)
Ongoing injury episode	
Yes	26 (19)
No	111 (81)
Self-rated health (point prevalence)	
Excellent	70 (51)
Good	62 (45)
Fair	5 (4)
Poor	0 (0)
International Obesity Task Force Body Mass Index (IOTF-BMI), n=132	
Underweight (IOTF-BMI ≤18.5)	16 (12)
Normal	111 (84)
Overweight (IOTF-BMI ≥25)	5 (4)
Menarche	
No	71 (52)
Yes	66 (48)
Menarcheal age (n=63)	
≤11 years	12 (19)
12 years	15 (24)
13 years	23 (37)
≥14 years	13 (21)
Irregular menstruation	
Yes	28 (20)
No/not menarche	109 (80)
Weight reduction behaviour (n=90)*	
No, my weight is fine	71 (79)
No, but I should lose some weight	11 (12)
No, because I need to put on weight	4 (4)
Yes	4 (4)
Weight concern (n=133)	
Yes, careful not to gain weight	39 (29)
Yes, careful not to lose weight	9 (7)
No, I do not care if I gain or lose weight	85 (64)
Body image perception (n=136)	
Underweight	10 (7)
Normal	97 (71)
Overweight	29 (21)
Asked to adjust weight	
Yes, to gain weight	13 (9)
Yes, to lose weight	10 (7)
No	114 (83)

*Question not asked to skaters <12 years.

### Anxiety and depression caseness

The mean short-STAI score among the skaters was 12.5 (SD 2.1), with 47% displaying anxiety caseness (score >12). The mean WHO-5-score was 62.6 (SD 17.4), with 10% of the skaters reporting scores indicative of depression caseness (score ≤50 for those ≥17 years, ≤40 for children 9–12 years, ≤36 for adolescents 13–16 years). About half (n=71, 52%) of the skaters reported neither anxiety nor depression caseness, 12 skaters (9%) reported scores indicating caseness in both categories, while 52 (38%) skaters reported only anxiety caseness and only 2 (1%) skaters reported only depression caseness.

### Factors associated with caseness

Anxiety caseness was in the simple binary logistic regression associated with older age, a previous or ongoing injury episode, the RED-S-index, skipping more weekly main meals, overweight body image perception and higher figure skating load. The determinants remaining in the multiple model were older age and overweight body image perception (table 3).

**Table 3:** Factors associated with anxiety caseness (short-STAI score >12) among participants (n=137)

	Simple models		Multiple model*		Multiple model: age excluded†		Multiple model: body image perception excluded‡		Multiple model: age and body image perception excluded§	
	OR (95% CI)	P value	OR (95% CI)	P value	OR (95% CI)	P value	OR (95% CI)	P value	OR (95% CI)	P value
Age (years)	1.3 (1.1 to 1.5)	<0.001	1.2 (1.1 to 1.4)	0.005			1.3 (1.1 to 1.5)	<0.001		
Previous or ongoing injury episode (No/Yes)	2.5 (1.2 to 5.0)	0.012							2.5 (1.2 to 5.0)	0.012
RED-S indicators (no/yes)	2.1 (1.0 to 4.5)	0.047								
Skipped main meals per week (continuous)	1.1 (1.0 to 1.3)	0.020					1.1 (1.0 to 1.3)	0.036		
Body image perception:										
Normal (reference category)		<0.001		0.003		<0.001				
Underweight	2.8 (0.7 to 10.7)	0.127	3.1 (0.8 to 12.1)	0.107	2.8 (0.7 to 10.7)	0.127				
Overweight	9.0 (3.2 to 25.8)	<0.001	5.9 (2.0 to 17.6)	0.001	9.0 (3.2 to 25.8)	<0.001				
Figure skating load (low/high)	2.1 (1.0 to 4.3)	0.041								
Mean weekly training hours:										
≤6 (reference category)		0.927								
7–12	1.1 (0.5 to 2.3)	0.771								
≥13	1.2 (0.4 to 3.9)	0.736								
Parental education level (high/low)	1.5 (0.8 to 3.0)	0.213								

*Nagelkerke R^2^=0.28.

†Nagelkerke R^2^=0.20.

‡Nagelkerke R^2^=0.20.

§Nagelkerke R^2^=0.06.

RED-S, Relative Energy Deficiency in Sport; STAI, State-Trait Anxiety Inventory.

When in the sensitivity analysis excluding age from the multiple model, only overweight body image perception was significantly associated with anxiety caseness (table 3). Excluding body image perception from the multiple model resulted in older age remaining in the model and skipping more main meals becoming significantly associated with anxiety caseness. A previous or ongoing injury episode was introduced as a determinant when excluding age and body image perception from the model. Nagelkerke R^2^ was reduced from 0.28 (age and body image perception in the model) and 0.20 (either age or body image perception in the model, respectively) to 0.06 when both variables were excluded from the model (table 3).

Skaters reporting neither anxiety nor depression caseness were younger than those reporting only anxiety caseness or both anxiety and depression caseness (table 4). The group with only depression caseness was too small (n=2) to be included in the comparative analysis.

**Table 4:** One-way analysis of variance (ANOVA) with post-hoc Bonferroni correction between participants' mean age difference in years (95% CI) between groups in relation to respondents reported short-STAI and WHO-5 scores (n=137)

	Participants n (%)	Age mean (SD)	Group comparison Mean age difference (95% CI) p value	
			Anxiety caseness+depression caseness	Anxiety caseness+no depression caseness
Anxiety caseness+depression caseness	12 (9)	15.4 (3.9)	–	–
No anxiety caseness+depression caseness	2 (2)	15.0 (−)*	†	†
Anxiety caseness+no depression caseness	52 (38)	13.8 (2.6)	−1.7 (−3.8 to 0.5) p=0.186	–
Neither anxiety nor depression caseness	71 (52)	11.9 (2.7)	−3.5 (−5.6 to −1.5) p<0.001	−1.9 (−3.1 to −0.7) p=0.001

Overall ANOVA p<0.001.

*SD could not be calculated.

†The group with only depression caseness was too small (n=2) to be included in the comparative analysis.

STAI, State-Trait Anxiety Inventory.

## Discussion

This study investigated the prevalence of anxiety and depression caseness among competitive female figure skaters and factors associated with anxiety and depression caseness. In the multiple model, overweight body image perception and older age were associated with anxiety caseness. Skaters reporting anxiety and depression caseness were older than those reporting no caseness and those reporting only anxiety caseness.

### Anxiety and depression caseness

The reported anxiety caseness prevalence of 47% can be compared with findings from similar studies. For example, 37% of European girls aged 15 years feel nervous more than once a week.^29^ The observed mean WHO-5 score of 62.6 is similar to scores found in adolescent European girls participating in sports (61.7 for individual sports and 63.7 for team sports, respectively).^30^ Participants with anxiety and depression caseness were older than those with only anxiety caseness and those with no reported caseness. These findings are consistent with previous research and increased prevalence rates of mental health conditions during ageing through adolescence.^14^ It could be that young female adolescents predisposed to anxiety stay longer in sports, which warrants further investigations into personality traits such as perfectionism and competitiveness in a figure skating population.

### Factors associated with anxiety caseness

We found a significant association between anxiety caseness and overweight body image perception in the sensitivity analysis when excluding the age variable from the multiple model. Correspondingly, age remained a significant explanatory factor when excluding overweight body image perception. This indicates a strong association between body image and reported anxiety caseness. Due to the cross-sectional study design, no conclusions can be drawn about the causality in the interaction between anxiety and body image. However, the association between overweight body image perception and anxiety caseness is concerning but not unexpected since body dissatisfaction in adolescence is associated with several psychiatric diagnoses.^31^ Furthermore, low body satisfaction during early and middle adolescence predicts later signs of more global mental distress (such as depressive symptoms and lower self-esteem).^32^ It may be associated with anxiety disorder symptoms.^33^ It also predicts weight-reduction behaviour.^31 34^

Our findings suggest that methods for identifying mental health problems at different levels need more attention. However, the observations made in this study should be interpreted in light that participating skaters rated their health more positively than the general population and were, to a lesser extent, engaging in weight reduction behaviours.^35^ Notably, the figure skaters in this study reported body dissatisfaction to a lesser extent than the general Swedish population reported, and they ate breakfast more often than their peers.^35^ It is notable that skaters, on the one hand, rate their health more positively than the general population. The majority report a normal IOTF-BMI, but at the same time, report a high prevalence of anxiety and depression caseness. Body image self-discrepancy has been proposed in early adolescence as a risk factor for depressive symptoms.^36^ In adolescents, older age has been found to moderate the link between physical activity and a body image perception of being overweight and between overweight perception and life satisfaction.^37^

Furthermore, it has recently been pointed out that the complexity of separating normal states related to performance issues vs a mental illness or mental disorders may not be sufficiently considered in current research.^38^ An individual may simultaneously express overall high well-being and symptoms of mental illness, such as anxiety.^39^ Increased but short-term stress reactions related to challenging sports situations, such as competitions or temporary setbacks, are a normal part of figure skating. For children and adolescents, such short-term stress reactions may also occur in relation to exams, for example, the national tests in school, which in Sweden takes place every spring. Fluctuations in mood may also be caused by training load, with a high training load associated with mood disturbances.^38^ Also, it is important to consider that caseness in terms of a screening condition that qualifies for psychiatric examination in no way equals a psychiatric diagnosis, which is a complex process.^13 40^

### Strengths and limitations

This is one of few studies targeting a total population of competitive figure skaters. The measures used (short-STAI and WHO-5) were chosen because they had been validated for all age groups (children, adolescents and adults) covered by this study, aiming to investigate the mental health of a community sample of Swedish competitive figure skaters. After this study was planned, additional measures for assessing anxiety and depression were published. For example, Gouttebarge *et al* presented a mental health screening measure validated for elite athletes 16 years or older.^41^ In this study, the response rate of 36% is a potential source of bias, and several other factors may also have impacted our findings. The cross-sectional design introduces a possible recall bias. Moreover, in this study, irregular menstruation was considered an RED-S indicator. However, irregular menstruation may occur for other reasons, such as in the years following menarche, endometriosis or polycystic ovary syndrome. This study defined anxiety caseness as reporting a short-STAI-score >12 based on the same principle used for the STAI-S.^20^ This was a semantically defined cut-off score with obvious face validity, but the authors have no empirical evidence regarding associations between reported anxiety caseness based on the cut-off score and downstream clinical illness. The anxiety caseness prevalence reported may be influenced by factors not measured in this study. For example, the timing of when the questionnaire’s administration may be important. The questionnaire was sent after the competitive season had ended, but during a time of the year when most Swedish schools have final exams, which may have influenced the reported anxiety and depression caseness levels. Also, those with the worst mental health may not have been represented in this study due to no longer participating in figure skating. Another limitation is that parents of skaters <12 years of age were advised to help interpret the questionnaire if needed. This might have affected the participants' willingness to fully disclose their symptoms and may have led to under-reporting symptoms among younger skaters. A randomised sample might be more representative of figure skaters in Sweden. However, the south-eastern region contains figure skaters of all levels and figure skating clubs of all sizes and conformities. Thus, we consider the results reasonably representative of young female Swedish figure skaters.

### Future research

Our findings have several implications for future research. First, girls and young women report more internalising symptoms such as anxiety and depression than their male counterparts. Therefore, comparisons with male figure skaters are warranted. Second, there is a gender imbalance with the under-representation of female athletes in research on sports and exercise psychology,^42^ highlighting the importance of future research on mental health among female athletes. Third, there have been several recent reports on mental abuse and an unhealthy sports culture in figure skating. Both have potentially negative impacts, ranging from injuries and disordered eating/eating disorders to anxiety, depression and suicidal ideation.^43^ Therefore, studies of the figure skating culture concerning abusive behaviour and its impact on physical and mental health among young figure skaters are warranted. Fourth, prospective studies are needed to identify causal risk factors associated with adverse mental health consequences such as anxiety, depression and eating disorders.

## Conclusion

Anxiety caseness was associated with overweight body image perception and older age in female competitive figure skaters. Older skaters reported generally worse mental health. More research on the mental health of figure skaters is warranted, considering comorbidity and focusing on those needing further assessment and support.

## Data Availability

Data are available on reasonable request. According to the Swedish Transparency and Secrecy Act, all filled-in questionnaires are public acts. The material may be shared on requests ensuring that the confidentiality demands can be handled.
