# Supplementary material for: A cross-sectional study of anxiety and depression caseness in female competitive figure skaters in Sweden
Source: BMJ Open Sport Exerc Med. 2023 Mar 8;9(1):e001491. doi: 10.1136/bmjsem-2022-001491 (PMC10008236; doi:10.1136/bmjsem-2022-001491)
Supplement: Supplementary data [file bmjsem-2022-001491supp001.pdf]

Supplementary Table I. Characteristics of participants (n=137).

	Participants n (%)
**The family's financial situation (n=90) †**	
Very good	54 (60)
Fairly good	30 (33)
Some or major financial difficulties	5 (6)
**Support from family and friends (n=90) †, ‡**	
My family really tries to help me	75 (83)
I get the emotional help and support I need from my family	68 (76)
I can talk about my problems with my family	64 (71)
My family is willing to help me make decisions	66 (73)
My friends really try to help me	61 (68)
I can count on my friends when things go wrong†	60 (67)
I have friends with whom I can share my joys and sorrows	69 (77)
I can talk about my problems with my friends	62 (69)
**Ever experienced bullying in connection with figure skating**	
Yes	14 (10)
No	123 (90)
**Skipping snacks on weekdays**	
Not skipping snacks on weekdays	98 (72)
≥1 weekday(s)	39 (28)
**Skipping snacks on weekends**	
Not skipping snacks on weekends	50 (36)
≥1 day(s) on weekends	87 (64)
**Eating fruits**	
≥1 time(s) per day	48 (35)
2-6 days per week	68 (50)
≤1 per week	21 (15)
**Eating vegetables**	
≥1 time(s) per day	79 (58)
2-6 days per week	45 (33)
≤1 per week	13 (9)
**Eating sweets**	
≥1 time(s) per day	5 (4)
2-6 days per week	85 (62)
≤1 per week	47 (34)
**Consuming sweet drinks (n=136)**	
≥1 time(s) per day	26 (19)
2-6 days per week	48 (35)
≤1 per week	63 (46)
**Use of dietary supplement (n=87) †**	
No	62 (71)
Yes	25 (29)
Kind of supplement used (multiple options possible) **(n=43)** †	
Energy drinks	5 (12)
Energy bars	17 (40)
Vitamin or mineral tablets	15 (35)
Other (nutritional drinks, protein powder, lactic acid tablets)	6 (14)

SD=Standard Deviation.
† Question not asked to skaters <12 years
‡ Multidimensional Perceived Social Support Scale (MPSS). Showing those answering a score of 6 or 7 (i.e., those that agree with the sentence).
